# Niche differentiation and biogeography of Bathyarchaeia in paddy soil ecosystems: a case study in eastern China

**DOI:** 10.1186/s40793-024-00555-8

**Published:** 2024-03-01

**Authors:** Xingyun Yi, Kristian Koefoed Brandt, Shudan Xue, Jingjing Peng, Yifei Wang, Meng Li, Ye Deng, Guilan Duan

**Affiliations:** 1 State Key Lab of Urban and Regional Ecology, Research Center for Eco-Environmental Sciences, Chinese Academy of Sciences, 18 Shuangqing Road, Haidian District, 100085 Beijing, China; 2 Department of Plant and Environmental Sciences, University of Copenhagen, Thorvaldsensvej 40, 1871 Frederiksberg, Denmark; 3 Sino-Danish Center (SDC), 101408 Beijing, China; 4 University of Chinese Academy of Sciences, 100049 Beijing, China; 5 College of Resources and Environmental Sciences, China Agricultural University, 10093 Beijing, China; 6 Archaeal Biology Center, Institute for Advanced Study, Shenzhen University, 518060 Shenzhen, Guangdong China; 7 Shenzhen Key Laboratory of Marine Microbiome Engineering, Institute for Advanced Study, Shenzhen University, 518060 Shenzhen, Guangdong China

**Keywords:** *Bathyarchaeia*, Paddy soils, Community assembly, Co-occurrence network

## Abstract

*Bathyarchaeia* (formerly *Bathyarchaeota*) is a group of highly abundant archaeal communities that play important roles in global biogeochemical cycling. *Bathyarchaeia* is predominantly found in sediments and hot springs. However, their presence in arable soils is relatively limited. In this study, we aimed to investigate the spatial distributions and diversity of *Bathyarchaeia* in paddy soils across eastern China, which is a major rice production region. The relative abundance of *Bathyarchaeia* among total archaea ranged from 3 to 68% in paddy soils, and *Bathy-6* was the dominant subgroup among the *Bathyarchaeia* (70–80% of all sequences). *Bathyarchaeia* showed higher migration ability and wider niche width based on the neutral and null model simulations. *Bathy-6* was primarily assembled by deterministic processes. Soil pH and C/N ratio were identified as key factors influencing the *Bathyarchaeia* composition, whereas C/N ratio and mean annual temperature influenced the relative abundance of *Bathyarchaeia*. Network analysis showed that specific *Bathyarchaeia* taxa occupied keystone positions in the archaeal community and co-occurred with some methanogenic archaea, including *Methanosarcina* and *Methanobacteria*, and ammonia-oxidizing archaea belonging to *Nitrososphaeria*. This study provides important insights into the biogeography and niche differentiation of *Bathyarchaeia* particularly in paddy soil ecosystems.

**Supplementary Information:**

The online version contains supplementary material available at 10.1186/s40793-024-00555-8.

## Introduction

Archaea drives a series of global biogeochemical cycling of carbon and nitrogen [1–3]. *Bathyarchaeia* belongs to the kingdom archaea, which was initially discovered in hot springs. They were previously placed in the *Miscellaneous Crenarchaeotal Group* (MCG) [4, 5]. *Bathyarchaeia* has been found in various other environments, including sediments, volcanic mud, termite guts, bioreactors, and soils [6–11]. *Bathyarchaeia* is highly abundant in marine sediments, making them one of the most abundant groups of microorganisms on the earth [12, 13]. However, previous studies on *Bathyarchaeia* have mainly focused on sediments, whereas the distribution of *Bathyarchaeia* in arable soils is not much studied.

To date, pure cultures of *Bathyarchaeia* have not been successfully isolated. However, cultivation-independent studies suggest that this group of organisms possesses high physiological and metabolic diversity [14]. Members of *Bathyarchaeia* can grow on different substrates, such as detrital proteins, polymeric carbohydrates, fatty acids, methane, and other organic matter based on genome sequences [15–17]. Four genomes of *Bathyarchaeia* were reconstructed from White Oak River sediments. They contained genes encoding enzymes involved in acetogenesis using the reductive acetyl-CoA pathway, indicating an anaerobic lifestyle [16]. Furthermore, some *Bathyarchaeia* members are likely to perform dissimilatory nitrite reduction to ammonium [16], and a possible role in methane metabolism has also been suggested [15]. A previous study reported that supplementing rice paddy soil with fulvic acid significantly increased the relative abundance of *Bathyarchaeia* [18]. Therefore, *Bathyarchaeia* may play a role in the biodegradation of humus, which is abundantly present in paddy soils due to the slow microbial decomposition of plant and animal residues under flooding conditions. Paddy soil is an active zone of global carbon and nitrogen cycling. Therefore, studying the distribution and activity of *Bathyarchaeia* can be important for food production and climate change regulation.

Previous phylogenetic studies have classified *Bathyarchaeia* into 25 subgroups [13, 19], and different subgroups exhibit different ecological functions and distribution. Therefore, elucidating the mechanisms underlying *Bathyarchaeia* biogeography and community assembly in paddy soils can help predict corresponding ecological processes. Generally, the microbial community assembly can be described using the Niche-based theory or Neutral-based theory [20, 21]. Niche-based approaches consider that the community structure is influenced primarily by deterministic processes such as environmental filtering and species interactions [22, 23]. For instance, previous studies have revealed that the specific *Bathyarchaeia* subgroups show niche differentiation and exhibit different habitat preferences. Members of *Bathy-6* grow in suboxic zones and sulfide-depleted shallow layers of sediments, whereas members of *Bathy-8* prefer deeper and anoxic layers [24]. Furthermore, *Bathy-8* is considered an indicator of saline environments [24], whereas *Bathy-11* and *Bathy-5* are indicators of freshwater environments [25]. Moreover, salinity and total organic matter (TOC) are crucial factors affecting the abundance and composition of the *Bathyarchaeia* community [8, 10, 11]. The neutral theory hypothesizes that all individuals are ecologically identical, and the community structure is primarily influenced by stochastic processes such as random death and dispersal [26, 27]. Stochastic processes play crucial roles in influencing microbial community structures in various environments [21, 28]. However, the assembly processes of the *Bathyarchaeia* community have garnered less attention in arable soils.

A recent global meta-analysis reported that *Bathyarchaeia* is globally distributed in paddy soils with high abundance, and the predominant subgroup is *Bathy-6* [29]. The meta-analysis showed that the mean annual precipitation and the mean annual temperature could be associated with the relative abundance of *Bathyarchaeia* and Bathyarchaeial community structure, respectively [29]. However, this meta-analysis had some limitations, such as the limited availability of soil physicochemical parameters data and distribution. Therefore, studying niche differentiation governed by soil type-related factors was difficult. Furthermore, it is important to note that this meta-analysis could potentially be affected by various additional factors associated with soil management practices, including irrigation, anthropogenic interventions like flooding, the specific growth stages of rice, and the absence of uniform approaches for soil sampling procedures (e.g., sampling depth), DNA extraction techniques, and primer selection for sequencing. Such disparities can introduce potential biases into the results. Consequently, our approach involved the sampling of paddy soils from contrasting pedoclimatic regions across eastern China, all at the same stage of rice growth. We then conducted an examination of their taxonomic composition through Illumina sequencing of the 16 S rRNA genes. The primary objectives of this study encompassed characterizing the composition and diversity of *Bathyarchaeia* in paddy soils across eastern China, exploring the mechanisms governing the assembly of *Bathyarchaeia* communities in paddy soil, and delving into the differentiation of ecological niches and potential ecological functions of *Bathyarchaeia* within paddy soils.

## Materials and methods

### Soil sampling and property measuring

**Fig. 1:**
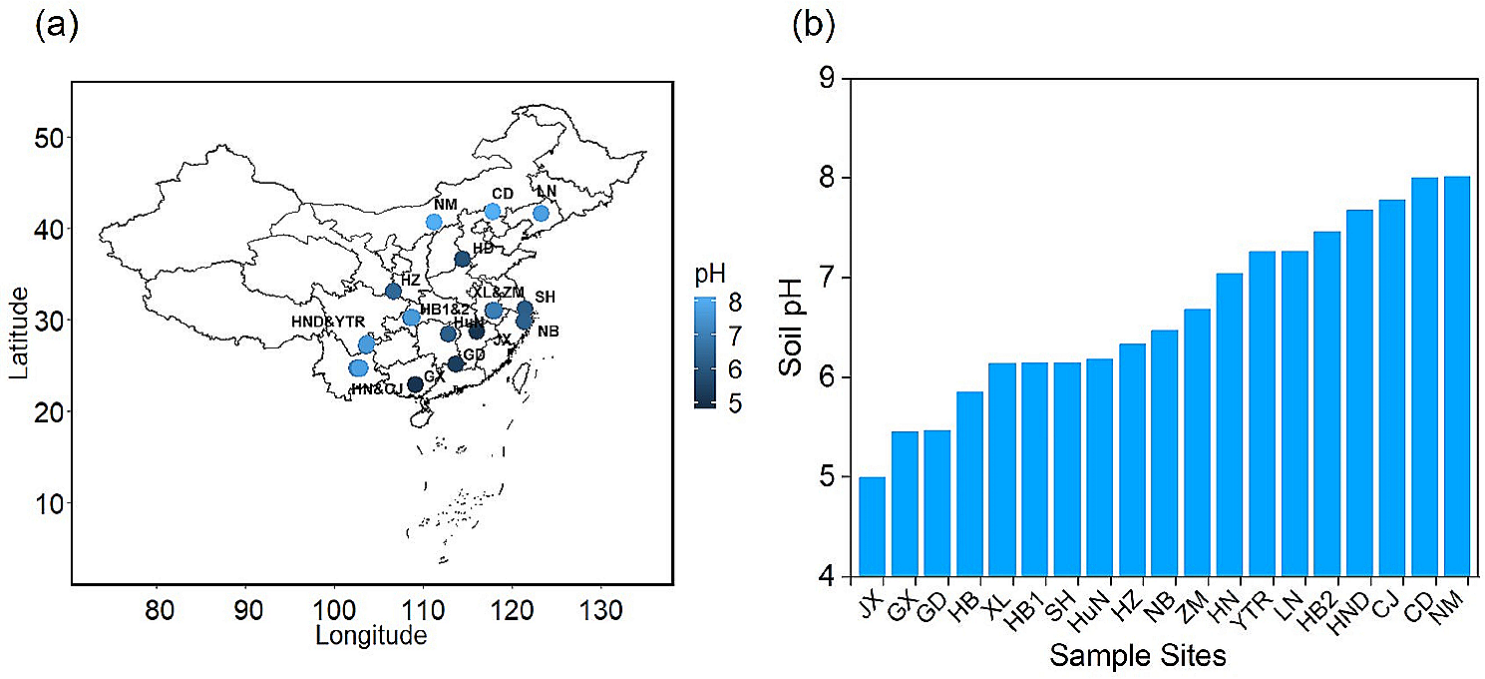
Paddy soils selected for the study. Map showing the geographical location of all sampling sites across eastern China (**a**). The color of the points represents the pH of the soil samples. pH of all the paddy soil samples (**b**)

Paddy soil samples (*n =* 57) with different chemical characteristics were collected from 19 sites (3 samples/site) in June 2020 across eastern China (Fig. 1a), which are the main rice-producing areas in China. While sampling, the paddy soils were flooded for over a month, with rice plants at the tillering stage. Three composite samples were collected from each site, indicating three replicates. These composites were created by combining five surface (< 10 cm) paddy soil cores. The samples were kept under ice and transported to the lab. Each soil sample was divided into the following two aliquots: one was freeze-dried for DNA extraction, and the other was stored at 4 °C for further soil chemical analysis.

Soil chemical parameters were evaluated, as reported previously [30]. Soil pH was detected with air-dried soil at a water ratio of 1:2.5 using a pH meter. Total carbon (TC) and total nitrogen (TN) were analyzed using an Elemental Analyzer (Vario EL, Elementar, Germany), and the resulting data was used for the calculation of the C/N ratio. Soil organic matter (SOM) was measured using a Muffle furnace (VULCAN 3–1750 A, NEYTECH, America). Nitrate (NO_3_^–^) and ammonium (NH_4_^+^) were analyzed using a Continuous Flow Analytical System (AA3, SEAL, Germany). Total iron (Fe) and manganese (Mn) analyses were performed using an Inductively Coupled Plasma Optical Emission Spectrometer (ICP-OES). The physicochemical properties of the soils are shown in Table S1. The soil properties differed significantly among different sites; for instance, the soil pH ranged from 4.8 to 8.1 (Fig. 1b), the C/N ratio ranged from 8.6 to 19.8, and the SOM content ranged from 2.81 to 39.37% (Table S1). Climatic indexes of different sites were obtained from the WorldClim database (www.worldclim.org), and the mean annual temperature (MAT) differed from 6.2 to 21.4 °C.

### DNA extraction and Illumina sequencing of the 16 S rRNA gene

DNA was extracted following the manufacturer’s protocol using the FastDNA SPIN kit (MP bio, CA, USA). The DNA was dissolved in 100 µL of sterilized deionized water and stored at − 18°C until further analysis. NanoDrop (ND-1000, USA) was used to detect DNA concentration and OD260/OD280. For the Illumina sequencing, the archaeal 16S rRNA genes were amplified using the primer pair 524F10extF/Arch958RmodR (5’-TGYCAGCCGCCGCGGTAA-3’/5’-YCCGGCGTTGAVTCCAATT-3’) [31], with 2 µL of DNA samples used as the template. Purified amplicons were sequenced using Illumina MiSeq PE300 (Illumina, San Diego, USA) by Majorbio Bio-Pharm Technology Co. Ltd. (Shanghai, China).

### Statistical analyses

Raw sequence data were analyzed using the R 4.0.1 platform using “dada2” packages for the analysis of the 16 S rRNA gene sequence (https://benjjneb.github.io/dada2/tutorial.html) [32]. Briefly, the adapters and primer sequences were first removed from raw sequence data using “cutadapat.” Moreover, clean sequences underwent trimming and merging. Amplicon sequence variants (ASVs) were derived following the removal of chimeric sequences, and their categorization was achieved using the Silva database release 138 to attain taxonomic insights [33, 34]. The ASV table was subsampled to the minimum requisite sequence count for subsequent statistical assessments. Calculation of α-diversity (Shannon and Chao1 indices) was executed with the “microeco” and “vegan” packages [35, 36]. The α-diversity and community composition visualizations were produced using Origin 2020 and the “ggplot2” packages in R [37]. Non-metric multidimensional scaling (NMDS) based on Bray-Curtis distances was performed using “micreco” packages to visualize the similarity between samples.

To analyze the community composition of *Bathyarchaeia*, a phylogenetic tree was constructed employing reference sequences from a prior study to classify the *Bathyarchaeia* subgroup [13]. The outgroup sequences belonged to *Crenarchaeum* (*Cenarchaeum symbiosum*) and *Nitrosoarchaeum* (*Nitrosoarchaeum koreensis*). These reference sequences encompassed 15 Bathyarchaeial subgroups [13]. ASVs affiliated with *Bathyarchaeia*, as per the Silva 138 database, were also selected. The construction of the phylogenetic tree was executed within the MEGA11 platform [38]. The alignment of all sequences was performed using ClustalW, and the Maximum Likelihood tree was employed for the construction, with a Bootstrap analysis (1000) being carried out to evaluate tree topology [13]. Based on the tree, the subgroup information of Bathyarchaeial ASVs was obtained and used for downstream statistical analyses. ArcMap software was used to predict and visualize the large-scale distribution pattern of *Bathy-6* across eastern China paddy soils for the analysis of predictive atlas maps. The Kriging interpolation method was used to estimate the relative abundance of *Bathy-6* across the whole map after the input of site information, including geographical coordinates and the relative abundance of *Bathy-6*. Further, the predictive maps were obtained using a province mask. For the heatmap of Bathyarchaeial ASVs, the figure was constructed using Evolview [39].

To investigate the determinism and stochasticity in influencing archaeal and Bathyarchaeial community structure, the Sloan neutral community model (NCM) was used to determine the effect of stochasticity on the archaeal and Bathyarchaeial community assembly using the “Hmisc” package [40, 41]. The “spaa” package was used to evaluate the width and overlap of the niche [42]. A cognitive assessment was employed to ascertain the connection between environmental factors and microbial communities with the utilization of the “linkET” package [43]. Structural equation modeling (SEM) was employed to quantify the direct and indirect influences of environmental factors on the shaping of both the archaeal and Bathyarchaeial communities, utilizing SPSS and AMOS software. To elucidate the correlational association between environmental factors and the relative abundance of Bathyarchaeial subgroups, Pearson’s correlation analysis was conducted through the “microeco” packages [35]. The graphical representations were generated using Origin 2020. For the co-occurrence network analysis, Spearman’s correlation coefficients between ASVs were initially calculated through the “microeco” packages on the R platform. The Spearman’s correlation threshold was set at a coefficient > 0.7 or < -0.7 with a significance level of *p* < 0.01. Subsequently, the networks were visualized using Gephi software.

### Availability of data and materials

The 16 S rRNA gene sequence data is deposited at the GenBank with BioProject accession number PRJNA1023015.

## Results

### Archaeal community composition and diversity

**Fig. 2:**
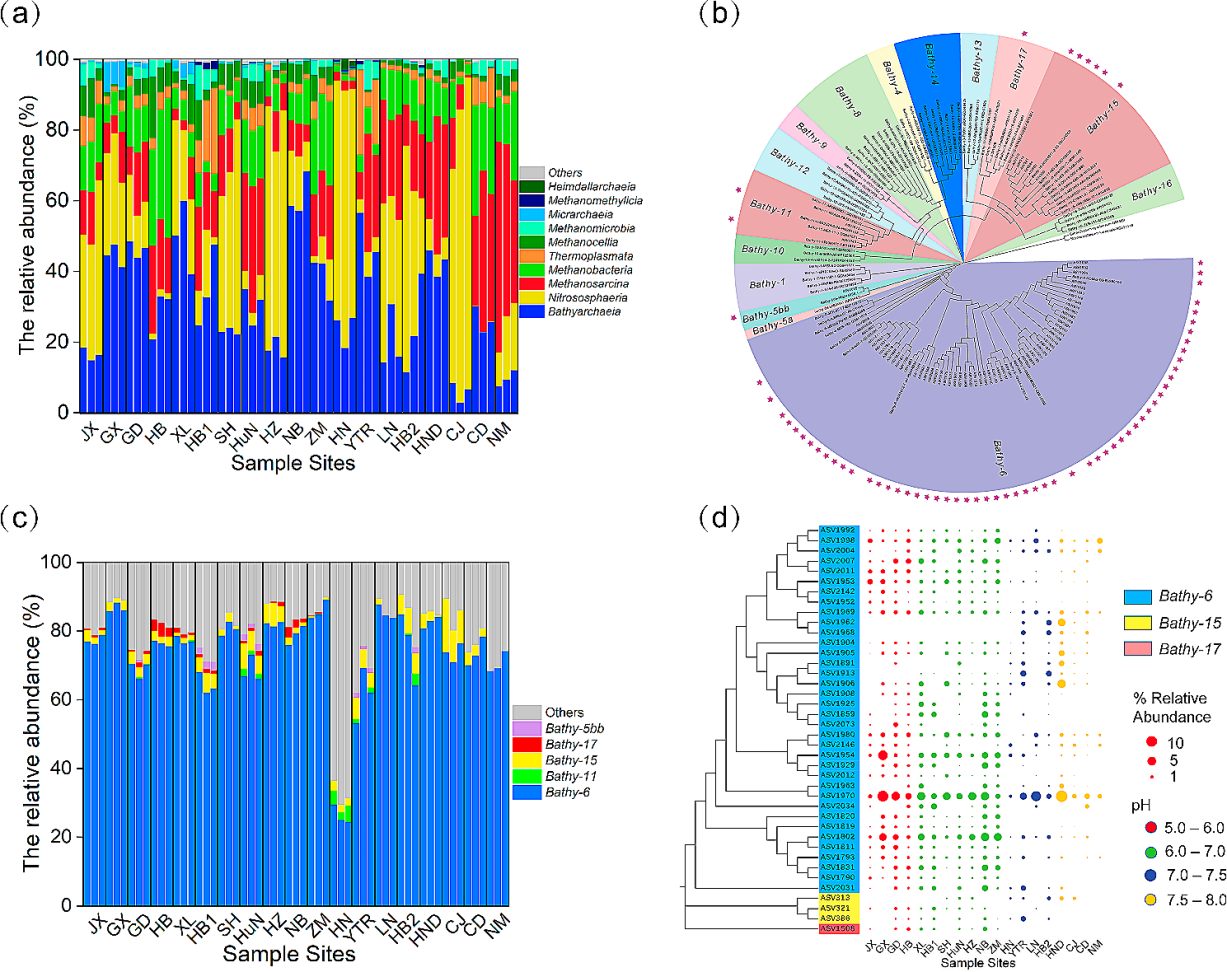
The composition and diversity of archaea and Bathyarchaeia community in paddy soils. The composition of the archaea community at class level (a). Phylogenetic tree of Bathyachaeial amplicon sequence variants (ASVs) (b). The star represents Bathyarchaeial ASVs from paddy soils in this study. The composition of the Bathyarchaeial subgroup in paddy soils (c). The heatmap of Bathyarchaeial ASVs (d). The 40 top abundant Bathyarchaeial ASVs were selected. The size of bubbles represents the relative abundance of ASVs. The color of the bubbles represents the pH of the soil samples. The color of ASV ID represents the subgroup of Bathyarchaeia

Archaeal α-diversity (Shannon and Chao1 indices) varied among different soil types (Fig. S1, *p* < 0.05). Generally, soils with medium pH (6.0 < pH < 7.0) showed higher archaeal α-diversity than soils with pH over 7.5. Archaeal community composition also differed between the different sites (Fig. S1). *Crenarchaeota* was the most abundant in most samples at the phylum level, accounting for 20–70% of total archaea, followed by *Halobacterota* (1–70%) as the second most abundant phylum (Fig. S2). The relative abundance of *Euryarchaeota* and *Thermoplasmatota* was 2–33% and 5–20%, respectively. *Asgardarchaeota* and *Micrarchaeota* were also detected in some samples with lower relative abundance. *Bathyarchaiea* and *Nitrososphaeria* were the two most dominant classes in most samples at the class level (Fig. 2a). However, the relative abundance of *Bathyarchaeia* differed drastically among different sites, ranging from 3 to 68% of total archaea. *Methanosarcina* and *Methanobacteria* were also detected at relatively high abundance in most samples, which belong to *Halobacterota* and *Euryarchaeota*, respectively.

### Distribution and diversity of *Bathyarchaeia* in paddy soils

The α-diversity of *Bathyarchaeia* in various paddy soils exhibited significant differences among distinct paddy soils, with the Shannon index ranging from 2.16 to 5.63 (refer to Fig. S3, *p* < 0.05). Notably, soils characterized by a pH > 7.5 displayed a reduced Bathyarchaeial α-diversity when compared to soils with a pH < 7.5. Furthermore, the NMDS analysis indicated that *Bathyarchaeia* within paddy soils clustered distinctly based on the sampling sites and pH (see Fig. S3).

Five subgroups (*Bathy-6*, *Bathy-11*, *Bathy-15*, *Bathy-17*, and *Bathy-5bb*) were identified within all the paddy soils (Fig. 2b). *Bathy-6* constituted the predominant subgroup across most samples, accounting for 70–80% of the total *Bathyarchaeia* (as depicted in Fig. 2c). Conversely, *Bathy-11*, *Bathy-17*, and *Bathy-5bb* were detected as less prevalent groups in numerous samples. It is noteworthy that paddy soils in Southeast China exhibited a higher relative abundance of *Bathy-6* in comparison to their counterparts in Northwest China (Fig. S4).

The heatmap, based on the 40 most abundant Bathyarchaeial ASVs, was generated with a focus on soil pH (as demonstrated in Fig. 2d). Out of the 40 most abundant Bathyarchaeial ASVs, 36 ASVs were affiliated with *Bathy-6*, while three ASVs were associated with *Bathy-15*, and one ASV was linked to *Bathy-17*. Furthermore, soil pH had a substantial impact on the distribution of these dominant ASVs (see Fig. 2d). Notably, two ASVs (ASV1970 and ASV1998) affiliated with *Bathy-6* were present at elevated levels in all the samples. Nevertheless, most other ASVs were solely detected in samples with acidic soil conditions (pH < 7.0).

### Bathyarchaeial community assembly in paddy soils

**Fig. 3:**
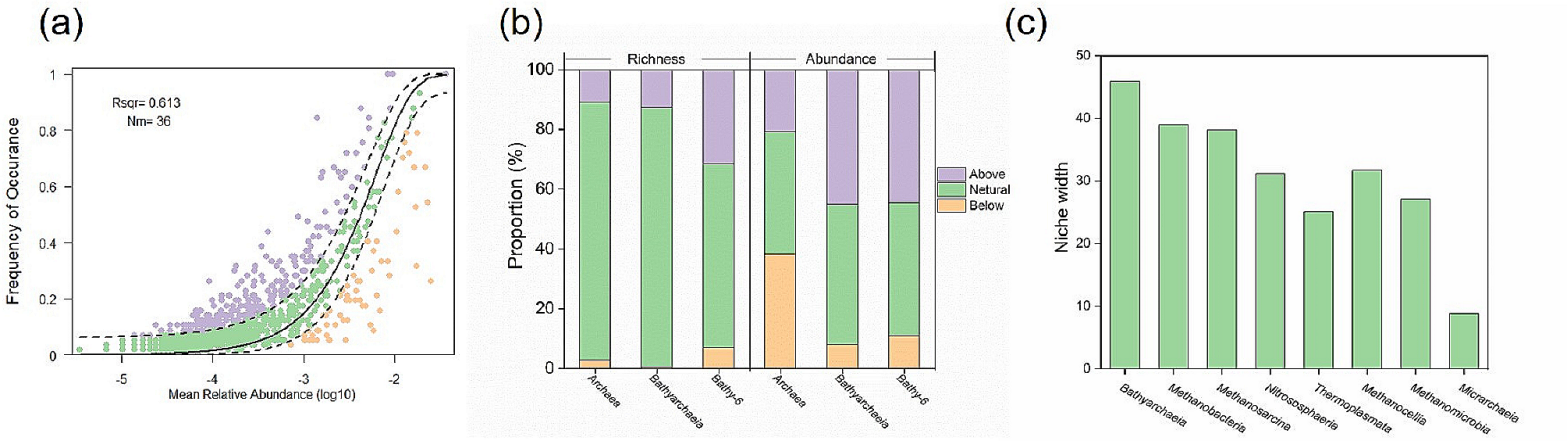
Community assembly progress of archaea and *Bathyarchaeia* in paddy soils. Neutral community model (NCM) of archaea (**a**). The solid blue lines mean the best fit to the NCM, and the dashed blue lines represent fit to the model under 95% confidence intervals. Nm means the metacommunity size times immigration, and R2 indicates the fit to this model. The proportions of richness (Amplicon sequence variants [ASV] number) and abundance (sequence number) of archaea and *Bathyarchaeia* based on the fitting to the model (**b**). Null model of Niche width of archaea at class level (**c**)

The NCM results at ASV levels indicated that stochastic processes were dominant in archaea community assembly, with an R^2^ value of 0.613 (Fig. 3a). We found that 86.2% of archaeal ASVs and 87.0% of Bathyarchaeial ASVs fitted to the neutral model, indicating that a majority of archaeal ASVs assembled in paddy soils following stochastic processes. However, the neutral model fitted ASVs accounted for only 40.9% and 47.0% relative abundance of total archaeal and Bathyarchaeial community, respectively. Approximately 12.6% of Bathyarchaeial ASVs deviated from the neutral expectation, accounting for more than 45.0% relative abundance. These results indicate that most low-abundance *Bathyarchaeia* assembled following stochastic processes, whereas high-abundance *Bathyarchaeia* mainly assembled following deterministic processes. This phenomenon can be further supported by the assembly process of the highest abundant *Bathy-6* (Fig. 2), exhibiting a higher percentage of the deterministic process (about 38.5%) than total archaea (13.8%) and total *Bathyarchaeia* (13%, Fig. 3b).

Among the 13% of Bathyarchaeial ASVs that diverged from neutral expectations, 12.6% were more frequently observed than anticipated by the NCM prediction, indicating that they exceeded the neutral prediction. ASVs exceeding the prediction are believed to possess greater migratory capabilities and a heightened ability to relocate to new habitats. Notably, *Bathy-6*, as previously mentioned, was the most prevalent Bathyarchaeial subgroup and exhibited a higher proportion of ASVs surpassing the NCM prediction compared to other archaea and *Bathyarchaeia*. This suggests that *Bathy-6* may have more robust migratory abilities and experience less constraint on dispersal than other subgroups. The breadth of ecological niches at the class level, as determined by the null model, was computed to assess the diversity of resource utilization among *Bathyarchaeia*. The outcomes demonstrated that the niche width of *Bathyarchaeia* in paddy soils surpassed that of other archaea, indicating a greater diversity in resource utilization among *Bathyarchaeia* in paddy soils (as visualized in Fig. 3c).

### Factors affecting the distribution of the Bathyarchaeial community

Our findings revealed that deterministic processes played a substantial role in the assembly of highly prevalent *Bathyarchaeia*, particularly the *Bathy-6* subgroup. This suggests that environmental filtering is a pivotal factor influencing their community structure. Consequently, we conducted an in-depth exploration of the relationship between environmental parameters and the distribution of *Bathyarchaeia* and *Bathy-6*.

**Fig. 4:**
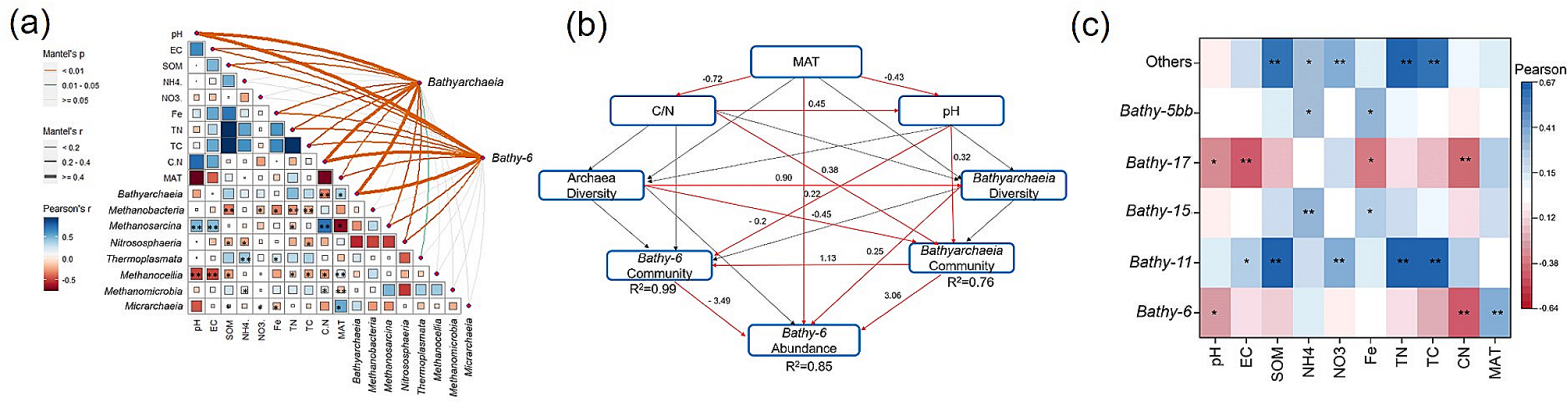
The effects of environmental parameters on the distribution of *Bathyarchaeia* and *Bathy-6*. The relationship between *Bathyarchaeia* and *Bathy-6* communities and environment parameters (**a**). SEM analyses reveal the direct and indirect effects of climate parameters, pH, and C/N on the relative abundance of *Bathy-6* (**b**). Red lines represent a significant correlation, and grey lines represent no significant correlation. R^2^ represents the proportion of variance explained. Pearson’s correlations between *Bathyarchaeia* and environment parameters at Subgroup level (**c**). * and ** represent the significance at 0.05 and 0.01 level

The statistical analysis demonstrated that the relative abundance of *Bathyarchaeia* exhibited a significant negative correlation with soil C/N and a positive correlation with the mean annual temperature (MAT) (as portrayed in Fig. 4a, *p* < 0.01). In the case of the Bathyarchaeial community, soil pH and C/N emerged as the two principal factors shaping the communities of *Bathyarchaeia* and *Bathy-6*. This observation was reinforced by the results of structural equation modeling (SEM), which assessed the direct and indirect impacts of environmental parameters on the Bathyarchaeial community and *Bathy-6* (Fig. 4b). SEM outcomes indicated that the *Bathy-6* community could be directly influenced by soil pH and the Bathyarchaeial community. Moreover, the relative abundance of *Bathy-6* was positively affected by MAT and indirectly influenced by soil C/N and pH.

Furthermore, we performed Pearson’s analysis to assess the influence of environmental parameters on the abundance of Bathyarchaeial subgroups detected in this study. The results indicated that soil EC, SOM, NH_4_^+^, NO_3_^–^, Fe, TN, and TC exhibited predominantly positive correlations with *Bath-5bb*, *Bathy-15*, *Bthy-11*, and negative correlations with *Bathy-17*. The relative abundance of *Bathy-6* displayed significant negative correlations with pH and C/N and positive correlations with MAT (Fig. 4c).

**Fig. 5:**
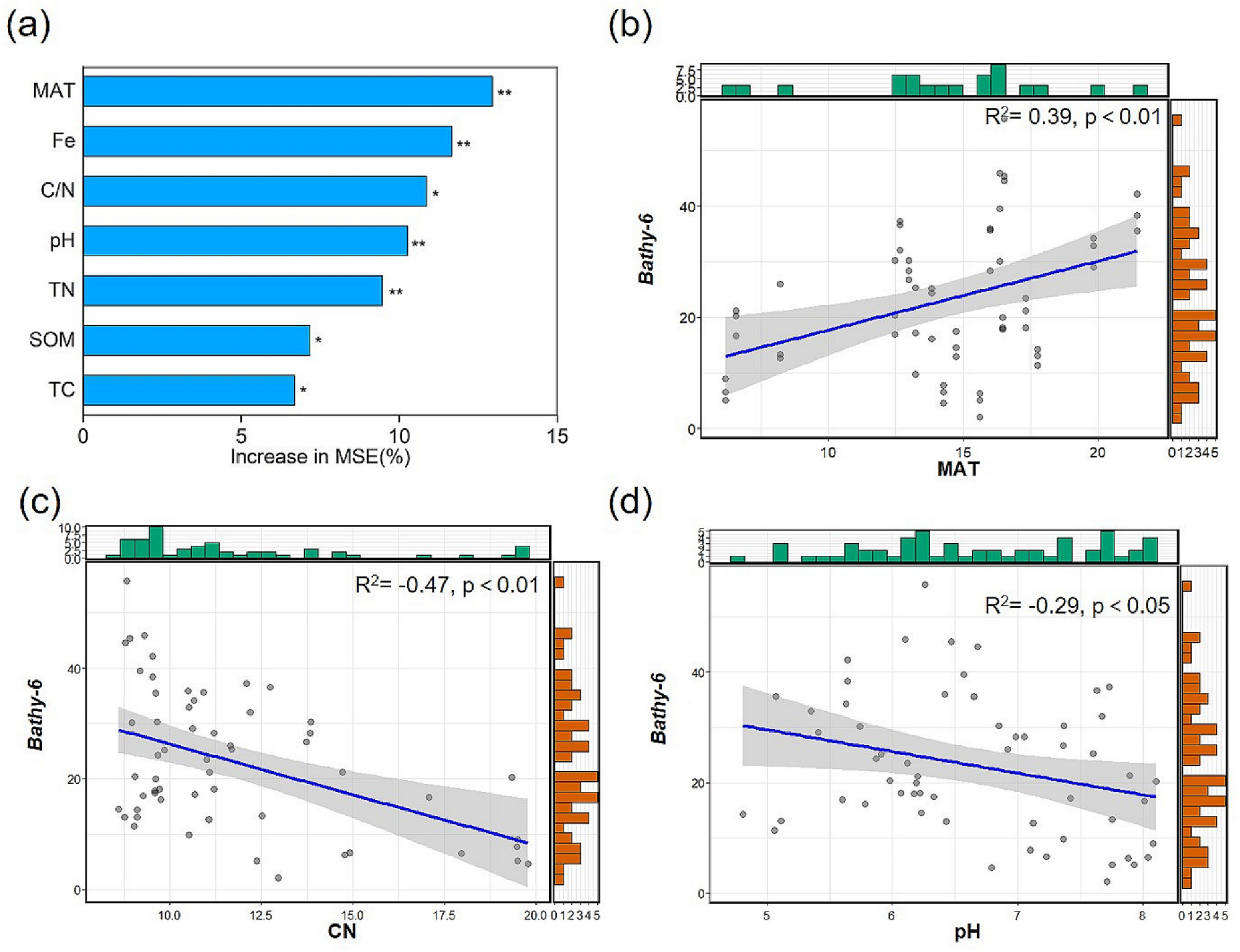
Drivers of distribution of *Bathy-6* were estimated. Environmental parameters predicting the relative abundance of *Bathy-6* according to Random Forest analysis (**a**). * and ** represent the significance at 0.05 and 0.01 level. The high value of the increase in MSE indicates more importance in the Random Forest Model. Linear least-squares regression analysis of the relative abundance of *Bathy-6* and MAT (**b**), C/N (**c**), and pH (**d**) was performed. The green and red bar charts overlaid on the axes represent the marginal distributions

Moreover, Random Forest analysis provided further insights, indicating that MAT, C/N, and pH are the primary factors driving the distribution of *Bathy-6* (Fig. 5). These results underscore that *Bathy-6* tends to have a higher abundance in environments characterized by higher temperatures, lower C/N ratios, and slightly acidic conditions (Fig. 5).

### Co-occurrence of *Bathyarchaeia* with soil microorganisms

**Fig. 6:**
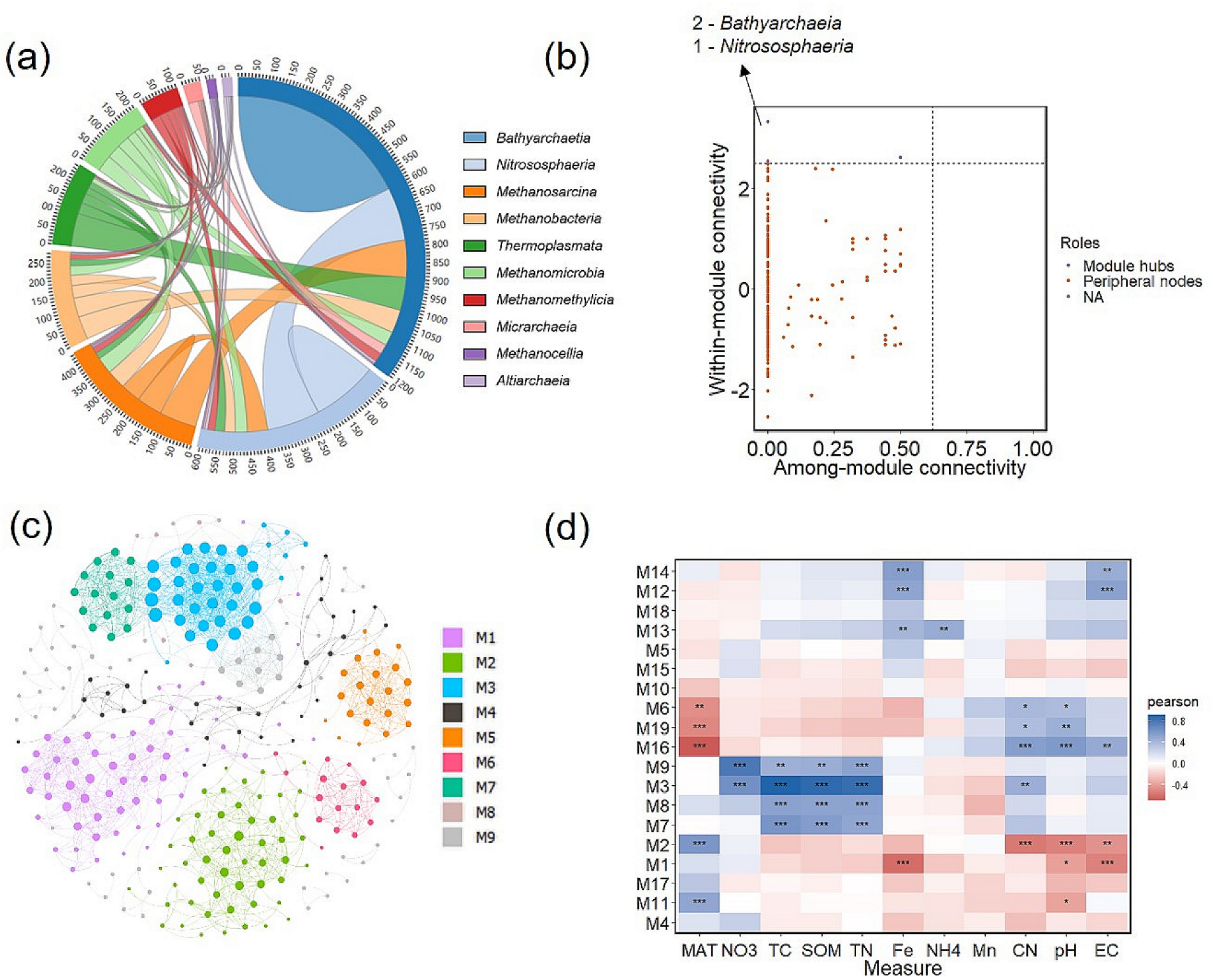
Co-occurrence network analyses of archaea and *Bathyarchaeia* in paddy soils. Co-occurrence network of archaea community based on the archaeal amplicon sequence variants (ASVs) with relative abundance higher than 1% (**a**). The numbers outside the circular plot represent the number of edges related with Class. Zi-Pi plot showing the distribution of archaeal ASVs based on their topological roles (**b**). Co-occurrence network of Bathyarchaeial community based on the Bathyarchaeial ASVs (**c**). Heatmap of the relationship between *Bathyarchaeia* modules and environmental parameters (**f**). * and ** represent the significance at 0.05 and 0.01 level

Co-occurrence network analysis was performed to determine the interactions of *Bathyarchaeia* with other archaea to examine the potential ecological functions of *Bathyarchaeia* in paddy soils. *Bathyarchaeia* mainly co-occurred with the members of *Nitrososphaeria*, *Methanosarcina*, and *Methanobacteria* (Fig. 6a). The co-occurred ASVs were selected to establish a phylogenetic tree (Fig. S5). The phylogenetic tree indicated that the co-occurrence archaea have various potential metabolisms (Fig. S5). Some co-occurrence archaea belonging to methanogens can use acetate and H_2_/CO_2_ to produce methane. The Zi-Pi plot indicated that *Bathyarchaeia* and *Nitrososphaeria* included keystone “species” (Fig. 6b). The keystone ASVs were ASV 1790 (*Bathyarchaeia*), ASV 2007 (*Bathy-6*), ASV 1248 (*Nitrososphaeria*), indicating their crucial role in maintaining the stability of microbial community structure. ASVs belonging to *Bathyarchaeia* were selected to establish the network (Fig. 6c). *Bathyarchaeia* and *Bathy-6* were divided into different modules. Different modules showed significant correlations with other environmental parameters, indicating a high diversity within *Bathyarchaeia* and *Bathy-6* in paddy soils (Fig. 6d). Niche overlap was evaluated to explore the interaction between *Bathyarchaeia* and other archaea. *Bathyarchaeia* showed a higher niche overlap with archaea, whereas *Nitrososphaeria* showed a lower niche overlap with archaea (Table S2).

## Discussions

### *Bathyarchaeia* inhabiting paddy soil is highly abundant but not diverse

*Bathyarchaeia* exhibits a widespread distribution in diverse environments. Past investigations have predominantly concentrated on marine, mangrove, and freshwater sediments, where *Bathyarchaeia* have been notably abundant [1, 8, 10, 24]. Nevertheless, the presence of *Bathyarchaeia* in arable soils has received limited scrutiny. Within this study, we have determined that the proportion of *Bathyarchaeia* relative to all archaea varies significantly among distinct paddy soils, constituting an average of approximately 31.2%. Concerning the community structure of *Bathyarchaeia* in paddy soils, the *Bathy-6* subgroup exclusively predominates across all surveyed sites, accompanied by a smaller representation of ASVs associated with Bathy*-11*, *Bathy-15*, *Bathy-5bb*, and *Bathy-17*. In comparison with our recent meta-analysis [29], the subgroup diversity of *Bathyarchaeia* in paddy soils within eastern China (comprising 5 subgroups) appeared lower than the global scale (comprising 7 subgroups), as *Bathy-18* and *Bathy-5b* were not detected in our study. For these rare Bathycechaeial subgroups in paddy soil in this study, they have been detected in various freshwater environment. *Bathy-18* and *Bathy-5bb* exhibited higher abundance in peatland with a pH ranging from 4.5 to 5 [44]. *Bathy-11* and *Bathy-15* showed higher abundance in in deeper sediments [44, 45]. They showed different niche preference. Furthermore, previous studies has also showed that *Bathyarchaeia* exhibit variable relative abundance in different paddy soils types [23, 46], highlighting the influence of pedoclimatic conditions on *Bathyarchaeia*. Therefore, it would be beneficial to explore the specific soil conditions that favor either low or high relative abundance of *Bathyarchaeia* in future research.

### Different assembly progress of abundant and rare bathyarchaeial taxa in paddy soils

NCM results indicated that stochastic processes dominated the archaea community assembly in paddy soils. The results from the NCM revealed that abundant *Bathyarchaeia* ASVs exhibit higher migratory capabilities within paddy soils and experience less dispersal limitation, implying a broader distribution range and a greater propensity for colonization within these soils [47]. The null model also indicated that *Bathyarchaeia* displays a wider niche breadth at the class level compared to other archaea in paddy soil. A broader niche breadth signifies that *Bathyarchaeia* possesses a heightened ability to adapt to various environments, accompanied by metabolic versatility, which in turn results in a widespread and abundant distribution pattern [28, 48, 49]. This characteristic is also supported by previous studies indicating that *Bathyarchaeia* were detected in various environments and were suggested to show multiple metabolisms. NCM results also showed that most low abundant *Bathyarchaeia* (87.0% of ASVs) fitted the neutral model. Soil nutrients in arable soils can decrease the effect of environmental filtering for microbial distribution [50]. Due to fertilization practice, paddy soils contain much higher nutrients than other environmental habitats, which increases the importance of stochastic processes in *Bathyarchaeia* community assembly and affects rare *Bathyarchaeia* taxa.

### Niche differentiation of *Bathyarchaeia* groups in paddy soils

To the best of our knowledge, this represents the inaugural study investigating the niche differentiation of *Bathyarchaeia* in relation to the physicochemical characteristics of arable soils, including pH and the C/N ratio. Our Random Forest analysis unveiled MAT as the preeminent factor influencing the abundance of *Bathyarchaeia* in paddy soil (as depicted in Fig. 5a). The relative abundance of *Bathyarchaeia* and *Bathy-6* exhibits a notably positive correlation with MAT (as seen in Fig. 4). This observation harmonizes with our recent global meta-analysis [29], underscoring the influence of temperature on *Bathyarchaeia* subgroups in soils, as similarly documented by previous study [9], who explored the impact of temperature on *Bathyarchaeia* subgroups in soils via multivariate regression tree analysis [9]. The discovery of *Bathyarchaeia* in hot springs further accentuates its remarkable adaptability to high-temperature environments [4].

We found the significance of the C/N ratio as an important factor in regulating the relative abundance of *Bathyarchaeia* in paddy soils (Figs. 4 and 5). However, in oligotrophic environments, such as sea and mangrove sediments, TOC was reported to be the major limiting factors for the abundance of *Bathyarchaeia*, regulating the quantity of *Bathyarchaeia* [8, 10]. However, in paddy soils, organic matter and ammonia are abundant due to fertilization; therefore, the C/N ratio becomes the major factor associated with the abundance of *Bathyarchaeia*. The dominant subgroup differs in sediments and paddy soils, causing a niche differentiation. These results can elaborate our understanding of the niche preference of *Bathyarchaeia* in different environments and give suggestions for the enrichment of *Bathyarchaeia*.

The results of Mantel analysis suggested that soil pH is also a key factor regulating the *Bathyarchaeia* and *Bathy-6* subgroups. SEM results further supported the important role of soil pH in influencing the community structure. The heatmap results also indicated that the abundance and number of ASVs also significantly correlate with soil pH. pH is a crucial factor in influencing bacterial and archaeal community structures in soils [23, 51], also influencing the community structure of *Bathyarchaeia* in mangrove sediments [8].

### *Bathyarchaeia* co-occurred with methanogens and ammonia oxidizers

Genomic analysis suggested that *Bathyarchaeia* may play a crucial role in global carbon and nitrogen cycling [13]. In this study, we found the interactions between *Bathyarchaeia* and other archaea were very complex, and *Bathyarchaeia* play an important role in the construction of the archaeal network (Fig. 5a). The co-occurrences of *Bathyarchaeia* and acetoclastic methanogens (i.e. *Methanosarcina*) have also been found in other environments [9, 11]. These results suggest that *Bathyarchaeia* can be involved in carbon cycling by producing acetate for acetoclastic methanogens. Co-occurrences of *Bathyarchaeia* and hydrogenotrophic methanogens such as *Methanobacteria* were also found in paddy soils in this study suggesting transfer of hydrogen from *Bathyarchaeia* to the methanogens.

*Bathyarchaeia* also frequently co-occurred with members of *Nitrososphaeria* (Fig. 5d), which is consistent with the results of the global meta-analysis [29]. Genes involved in ammonia and urea production were found in Bathyarchaeial MAGs, suggesting *Bathyarchaeia* serve as a transfer station for nitrogen compounds in the global nitrogen cycle [17]. Moreover, the metagenomic analysis suggested that *Nitrososphaeria* could use ammonia and urea [52]. Ammonia and urea might the bridges link specific *Bathyarchaeia* and *Nitrososphaeria* taxa [29]. However, as two dominant classes in the soil, their relative abundances exhibit a negative correlation in Fig. 4a. *Bathyarchaeia* and *Nitrososphaera* both play roles in nitrogen cycling, their specific ecological niches and metabolic activities likely determine their interactions in complex microbial communities. Agricultural systems depend on significant nitrogen fertilizer inputs for farm yield; therefore, the role of *Bathyarchaeia* in agricultural soils on the nitrogen cycle and factors influence the relationship between *Bathyarchaeia* and *Nitrososphaeria* warrants further research.

We also found that *Bathyarchaeia* indicated higher niche overlap with other archaea. Higher overlap means higher association with other microorganisms, whereas no higher competition was found in paddy soils. This view is supported by network analysis that *Bathyarchaeia* plays a crucial role in the structure of a network [53, 54]. In the future, more research is needed to investigate the ecological function of *Bathyarchaeia* in paddy soils.

### *Bathy-6* is the dominant subgroup in paddy soils with broad environmental adaptation

*Bathy-6* exhibits a widespread presence in terrestrial environments, including soil, freshwater sediments, and mangrove sediments [8, 9, 11], although it has also been detected in certain marine sediments [55]. In numerous prior studies, *Bathyarchaeia* were primarily observed in anaerobic sediments, consistent with their assumed anaerobic lifestyle [13]. Notably, Lazar et al. identified genes encoding enzymes responsible for responding to oxidative stress in *Bathy-6*, suggesting that *Bathy-6* members possess an ability to adapt to fluctuations in oxygen levels [16]. Additionally, Pan et al. reported the presence of oxygen-dependent metabolic pathways within certain *Bathy-6* genomes, hinting at a microaerophilic lifestyle for *Bathy-6* [17]. In our study, the results underscored that *Bathy-6* stands as the predominant subgroup within the *Bathyarchaeia* in all the paddy soils (see Fig. 3a). Flooded paddy soils are characterized by microaerophilic conditions due to the presence of dissolved oxygen in soil porewater and oxygen released by rice roots [56, 57]. This microoxic nature of paddy soil could elucidate why the anaerobic *Bathyarchaeia* subgroups are less prevalent, while *Bathy-6* becomes dominant in these environments. Collectively, these findings indicate that *Bathy-6* possesses wide-ranging environmental adaptability, accommodating both microoxic and anaerobic conditions. This suggests that *Bathy-6* may potentially play a role in the evolutionary transition of life from anaerobic to aerobic environments. Further investigations are necessary to unravel the mechanisms underpinning the high prevalence of Bathy-6 in paddy soils.

Furthermore, beyond adapting to diverse oxygen conditions, certain *Bathy-6* ASVs exhibit the capability to thrive across a broad pH range from 5 to 8. *Bathy-6* demonstrates resilience not only in oligotrophic marine sediments but also in eutrophic paddy soils with elevated TC. Our network analysis indicated that *Bathy-6* members are subdivided into several groups. These results collectively underscore that *Bathy-6* possesses versatile metabolic capabilities, thrives in diverse habitats, and exhibits varied lifestyles, consistent with genomic predictions [17].

## Conclusions

Our results extend our knowledge on *Bathyarchaeia* niche differentiation in paddy soils. *Bathyarchaeia* was shown to be highly abundant in most paddy soils across eastern China, which were predominated by *Bathy-6*. The abundance and diversity of *Bathyarchaeia* varied considerably among different soils. The relative abundance of *Bathyarchaeia* in paddy soils was linked to soil C/N ratio and MAT, whereas soil pH was the key factor influencing *Bathyarchaeia* community structure. *Bathy-6* is assembled mainly by deterministic progress with higher relative abundance in high temperature, low C/N, and slightly acidic environments. The network analysis revealed that *Bathyarchaeia* helps in maintaining the stability of archaeal community structure, with high co-occurrence with acetoclastic methanogens, some hydrogenotrophic methanogens, and archaeal ammonia oxidizers. Further research is required to reveal the specific functions of these organisms in paddy soils.

### Electronic supplementary material

Below is the link to the electronic supplementary material.

**Additional file 1:** It’s.xlsx file and include two tables. The title of the tables are Physicochemical parameter details of the samples (Table S1) and Nice overlap of Bathyarchaeia and other archaea (Table S2), respectively.

**Additional file 2:** It’s.docx file and include five figures. The title of the figures are The archaeal diversity of paddy soil (Fig. S1), The archaeal community in paddy soils at the phylum level (Fig. S2), The Bathyarchaeial diversity of paddy soil (Fig. S3), The atlas maps predicted the distribution of *Bathy-6* across paddy soils (Fig. S4) and Phylogenetic tree of ASVs co-occurrence with *Bathyarchaeia* (Fig. S5).

## Data Availability

The datasets analysed during the current study are available in the GenBank repository, https://www.ncbi.nlm.nih.gov/bioproject/PRJNA1023015/.

## Abbreviations

ASV: Amplicon sequence variant
TC: Total carbon
TN: Total nitrogen
MAT: Mean annual temperature
NMDS: Non-metric multidimensional scaling
NCM: Neutral community model
SEM: Structural equation modeling

## References

[1] Baker BJ, De Anda V, Seitz KW, Dombrowski N, Santoro AE, Lloyd KG (2020). Diversity, ecology and evolution of Archaea. Nat Microbiol.

[2] Bates ST, Berg-Lyons D, Caporaso JG, Walters WA, Knight R, Fierer N (2011). Examining the global distribution of dominant archaeal populations in soil. Isme J.

[3] Offre P, Spang A, Schleper C (2013). Archaea in Biogeochemical cycles. Annu Rev Microbiol.

[4] Barns SM, Delwiche CF, Palmer JD, Pace NR (1996). Perspectives on archaeal diversity, thermophily and monophyly from environmental rRNA sequences. P Natl Acad Sci USA.

[5] Inagaki F, Suzuki M, Takai K, Oida H, Sakamoto T, Aoki K (2003). Microbial communities associated with geological horizons in coastal subseafloor sediments from the Sea of Okhotsk. Appl Environ Microb.

[6] Calusinska M, Goux X, Fossepre M, Muller EEL, Wilmes P, Delfosse P. A year of monitoring 20 mesophilic full-scale bioreactors reveals the existence of stable but different core microbiomes in bio-waste and wastewater anaerobic digestion systems. Biotechnol Biofuels. 2018;11. 10.1186/s13068-018-1195-8 PMC6052691 30038663

[7] Loh HQ, Herve V, Brune A. Metabolic potential for reductive acetogenesis and a Novel Energy-converting [NiFe] Hydrogenase in Bathyarchaeia from Termite guts - A Genome-Centric analysis. Front Microbiol. 2021;11. 10.3389/fmicb.2020.635786 PMC7886697 33613473

[8] Pan J, Chen YL, Wang YM, Zhou ZC, Li M (2019). Vertical distribution of Bathyarchaeotal communities in Mangrove wetlands suggests distinct Niche preference of Bathyarchaeota Subgroup 6. Microb Ecol.

[9] Xiang X, Wang RC, Wang HM, Gong LF, Man BY, Xu Y. Distribution of Bathyarchaeota communities across different terrestrial settings and their potential ecological functions. Sci Rep-Uk. 2017;7. 10.1038/srep45028 PMC5359579 28322330

[10] Yu TT, Liang QY, Niu MY, Wang FP (2017). High occurrence of Bathyarchaeota (MCG) in the deep-sea sediments of South China Sea quantified using newly designed PCR primers. Env Microbiol Rep.

[11] Zou DY, Pan J, Liu ZB, Zhang CL, Liu HB, Li M. The distribution of in Surface sediments of the Pearl River Estuary along Salinity Gradient. Front Microbiol. 2020;11. 10.3389/fmicb.2020.00285 PMC7056671 32174899

[12] He Y, Li M, Perumal V, Feng X, Fang J, Xie J et al. Genomic and enzymatic evidence for acetogenesis among multiple lineages of the archaeal phylum Bathyarchaeota widespread in marine sediments. Nat Microbiol. 2016;1(6). 10.1038/nmicrobiol.2016.35 27572832

[13] Zhou Z, Pan J, Wang F, Gu J-D, Li M (2018). Bathyarchaeota: globally distributed metabolic generalists in anoxic environments. FEMS Microbiol Rev.

[14] Lewis WH, Tahon G, Geesink P, Sousa DZ, Ettema TJG (2021). Innovations to culturing the uncultured microbial majority. Nat Rev Microbiol.

[15] Evans PN, Parks DH, Chadwick GL, Robbins SJ, Orphan VJ, Golding SD (2015). Methane metabolism in the archaeal phylum Bathyarchaeota revealed by genome-centric metagenomics. Science.

[16] Lazar CS, Baker BJ, Seitz K, Hyde AS, Dick GJ, Hinrichs KU (2016). Genomic evidence for distinct carbon substrate preferences and ecological niches of Bathyarchaeota in estuarine sediments. Environ Microbiol.

[17] Pan J, Zhou ZC, Beja O, Cai MW, Yang YC, Liu Y et al. Genomic and transcriptomic evidence of light-sensing, porphyrin biosynthesis, Calvin-Benson-Bassham cycle, and urea production in Bathyarchaeota. Microbiome. 2020;8(1). 10.1186/s40168-020-00820-1 PMC7110647 32234071

[18] Yi XY, Yang YP, Yuan HY, Chen Z, Duan GL, Zhu YG (2019). Coupling metabolisms of arsenic and iron with humic substances through microorganisms in paddy soil. J Hazard Mater.

[19] Rinke C, Chuvochina M, Mussig AJ, Chaumeil PA, Davin AA, Waite DW (2021). A standardized archaeal taxonomy for the genome taxonomy database. Nat Microbiol.

[20] Hanson CA, Fuhrman JA, Horner-Devine MC, Martiny JBH (2012). Beyond biogeographic patterns: processes shaping the microbial landscape. Nat Rev Microbiol.

[21] Zhou JZ, Ning DL. Stochastic Community Assembly: does it Matter in Microbial Ecology? Microbiol Mol Biol R. 2017;81(4). 10.1128/MMBR.00002-17 PMC5706748 29021219

[22] Chase JM, Myers JA (2011). Disentangling the importance of ecological niches from stochastic processes across scales. Philos T R Soc B.

[23] Jiao S, Xu YQ, Zhang J, Lu YH. Environmental filtering drives distinct continental atlases of soil archaea between dryland and wetland agricultural ecosystems. Microbiome. 2019;7. 10.1186/s40168-019-0630-9 PMC6359761 30709414

[24] Lazar CS, Biddle JF, Meador TB, Blair N, Hinrichs KU, Teske AP (2015). Environmental controls on intragroup diversity of the uncultured benthic archaea of the miscellaneous crenarchaeotal group lineage naturally enriched in anoxic sediments of the White Oak River estuary (North Carolina, USA). Environ Microbiol.

[25] Fillol M, Auguet JC, Casamayor EO, Borrego CM (2016). Insights in the ecology and evolutionary history of the Miscellaneous Crenarchaeotic Group lineage. Isme J.

[26] Hubbell SP, Borda-De-Agua L (2004). The unified neutral theory of biodiversity and biogeography: reply. Ecology.

[27] Tilman D (2004). Niche tradeoffs, neutrality, and community structure: a stochastic theory of resource competition, invasion, and community assembly. P Natl Acad Sci USA.

[28] Chen SM, Waghmode TR, Sun RB, Kuramae EE, Hu CS, Liu BB. Root-associated microbiomes of wheat under the combined effect of plant development and nitrogen fertilization. Microbiome. 2019;7(1). 10.1186/s40168-019-0750-2 PMC6806522 31640813

[29] Xue S-D, Yi X-Y, Cui H-L, Li M, Peng J-J, Zhu Y-G (2023). Global biogeographic distribution of Bathyarchaeota in paddy soils. mSystems.

[30] Yuan H-Y, Ding L-J, Wang N, Chen S-C, Deng Y, Li X-M (2016). Geographic distance and amorphous iron affect the abundance and distribution of Geobacteraceae in paddy soils in China. J Soils Sediments.

[31] Liu C, Li H, Zhang YY, Si DD, Chen QW (2016). Evolution of microbial community along with increasing solid concentration during high-solids anaerobic digestion of sewage sludge. Bioresource Technol.

[32] Callahan BJ, McMurdie PJ, Rosen MJ, Han AW, Johnson AJA, Holmes SP (2016). DADA2: high-resolution sample inference from Illumina amplicon data. Nat Methods.

[33] Quast C, Pruesse E, Yilmaz P, Gerken J, Schweer T, Yarza P (2013). The SILVA ribosomal RNA gene database project: improved data processing and web-based tools. Nucleic Acids Res.

[34] Yilmaz P, Parfrey LW, Yarza P, Gerken J, Pruesse E, Quast C (2014). The SILVA and all-species living Tree Project (LTP) taxonomic frameworks. Nucleic Acids Res.

[35] Liu C, Cui YM, Li XZ, Yao MJ. Microeco: an R package for data mining in microbial community ecology. FEMS Microbiol Ecol. 2021;97(2). 10.1093/femsec/fiaa255 33332530

[36] Oksanen JSG, Blanchet F, Kindt R, Legendre P, Minchin P, O’Hara R, Solymos P, Stevens MSE, Wagner H, Barbour M, Bedward M, Bolker B, Borcard D, Carvalho G, Chirico M, De, Caceres MDS, Evangelista H, FitzJohn R, Friendly M, Furneaux B, Hannigan G, Hill M, Lahti LMD, Ouellette M, Ribeiro Cunha E, Smith T, Stier A, Ter Braak C, Weedon J. vegan: Community Ecology Package_. R package version 2.6-4. 2022.

[37] Pingram MA, Collier KJ, Hamer MP, David BO, Catlin AK, Smith JP (2019). Improving region-wide ecological condition of wadeable streams: risk analyses highlight key stressors for policy and management. Environ Sci Policy.

[38] Tamura K, Stecher G, Kumar S (2021). MEGA11 Molecular Evolutionary Genetics Analysis Version 11. Mol Biol Evol.

[39] Subramanian B, Gao SH, Lercher MJ, Hu SN, Chen WH (2019). Evolview v3: a webserver for visualization, annotation, and management of phylogenetic trees. Nucleic Acids Res.

[40] Sloan WT, Lunn M, Woodcock S, Head IM, Nee S, Curtis TP (2006). Quantifying the roles of immigration and chance in shaping prokaryote community structure. Environ Microbiol.

[41] Harrell F Jr, Dupont C. Hmisc: Harrell Miscellaneous. R Package Version 4.2-0. 2019.

[42] Zhang Jspaa. SPecies Association Analysis_. R package version 0.2.2. 2016.

[43] Huang H. LinkET: everything is linkable. R package version 00. 2021;2.

[44] Xiang X, Wang HM, Man BY, Xu Y, Gong LF, Tian W, Yang H (2023). Diverse bathyarchaeotal lineages dominate archaeal communities in the Acidic Dajiuhu Peatland, Central China. Microb Ecol.

[45] Liu JW, Yang HM, Zhao MX, Zhang XH (2014). Spatial distribution patterns of benthic microbial communities along the Pearl Estuary, China. Syst Appl Microbiol.

[46] Zheng F, Chen Y, Xie W, Chen S, Liu H, Phelps TJ (2019). Diverse biological sources of core and intact polar isoprenoid GDGTs in terrace soils from southwest of China: implications for their use as environmental proxies. Chem Geol.

[47] Chen WD, Wen DH. Archaeal and bacterial communities assembly and co-occurrence networks in subtropical mangrove sediments under Spartina alterniflora invasion. Environ Microbiome. 2021;16(1). 10.1186/s40793-021-00377-y PMC8091715 33941277

[48] He ZB, Liu D, Shi Y, Wu XJ, Dai YX, Shang YW et al. Broader environmental adaptation of rare rather than abundant bacteria in reforestation succession soil. Sci Total Environ. 2022;828. 10.1016/j.scitotenv.2022.154364 35288131

[49] Pandit SN, Kolasa J, Cottenie K (2009). Contrasts between habitat generalists and specialists: an empirical extension to the basic metacommunity framework. Ecology.

[50] Shu DT, Guo YQ, Zhang BG, Zhang CF, Van Nostrand JD, Lin YB et al. Rare prokaryotic sub-communities dominate the complexity of ecological networks and soil multinutrient cycling during long-term secondary succession in China’s Loess Plateau. Sci Total Environ. 2021;774. 10.1016/j.scitotenv.2021.145737 33611012

[51] Tripathi BM, Stegen JC, Kim M, Dong K, Adams JM, Lee YK (2018). Soil pH mediates the balance between stochastic and deterministic assembly of bacteria. Isme J.

[52] Zhao J, Huang LB, Chakrabarti S, Cooper J, Choi E, Ganan C, Tolchinsky B, Triplett EW, Daroub SH, Martens-Habbena W (2023). Nitrogen and phosphorous acquisition strategies drive coexistence patterns among archaeal lineages in soil. Isme J.

[53] Deboer WF, Prins HHT. Large herbivores that strive mightily but eat and drink as friends.Oecologia. 1990;82(2):264–74. 10.1007/BF00323544 28312674

[54] Holt RD (1987). On the relation between niche overlap and competition– the effect of incommensurable niche dimensions. Oikos.

[55] Yu TT, Wu WC, Liang WY, Lever MA, Hinrichs KU, Wang FP (2018). Growth of sedimentary on lignin as an energy source. P Natl Acad Sci USA.

[56] Chen XP, Zhu YG, Xia Y, Shen JP, He JZ (2008). Ammonia-oxidizing archaea: important players in paddy rhizosphere soil?. Environ Microbiol.

[57] Li Y, Zhang Y, Hu J, Shen Q (2007). Contribution of nitrification happened in rhizospheric soil growing with different rice cultivars to N nutrition. Biol Fertil Soils.

