# Supplementary material for: Niche differentiation and biogeography of Bathyarchaeia in paddy soil ecosystems: a case study in eastern China
Source: Environ Microbiome. 2024 Mar 1;19:13. doi: 10.1186/s40793-024-00555-8 (PMC10908009; doi:10.1186/s40793-024-00555-8)
Supplement: Supplementary file 2 — Additional file 2: It’s.docx file and include five figures. The title of the figures are The archaeal diversity of paddy soil (Fig. S1), The archaeal community in paddy soils at the phylum level (Fig. S2), The Bathyarchaeial diversity of paddy soil (Fig. S3), The atlas maps predicted the distribution of Bathy-6 across paddy soils (Fig. S4) and Phylogenetic tree of ASVs co-occurrence with Bathyarchaeia (Fig. S5). [file 40793_2024_555_MOESM2_ESM.docx]

**Supplementary material**
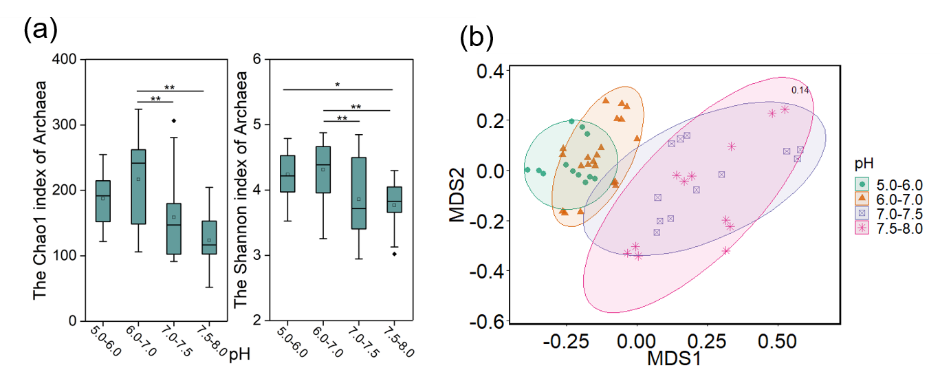


**Fig. S1.** The archaeal diversity of paddy soil. The α-diversity of archaea (a) and NMDS analyses of archaea based on ASV level with Bray-Curtis distances (b). * and ** represent the significance at 0.05 and 0.01 level.





**Fig. S2.** The archaeal community in paddy soils at the phylum level.


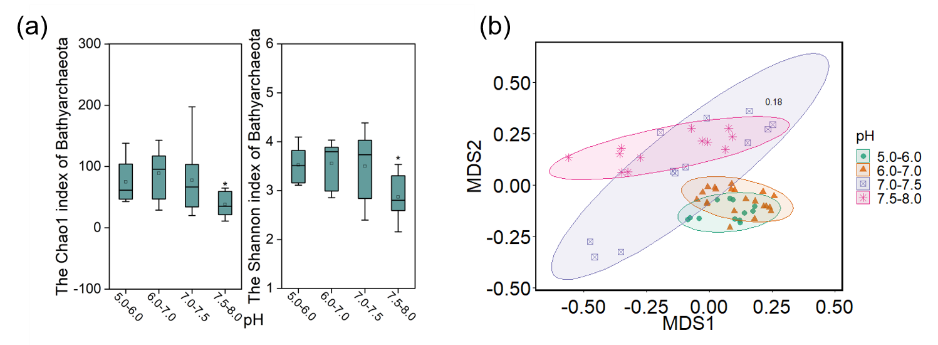


**Fig. S3.** The Bathyarchaeial diversity of paddy soil. The α-diversity of *Bathyarchaeia* (a) and NMDS analyses of *Bathyarchaeia* based on ASV level with Bray-Curtis distances (b). * represent the significance at 0.05 level.


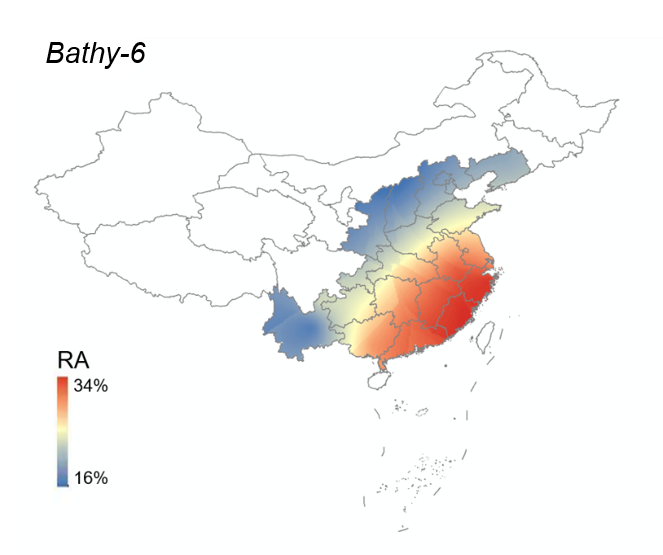


**Fig. S4.** The atlas maps predicted the distribution of *Bathy-6* across paddy soils.


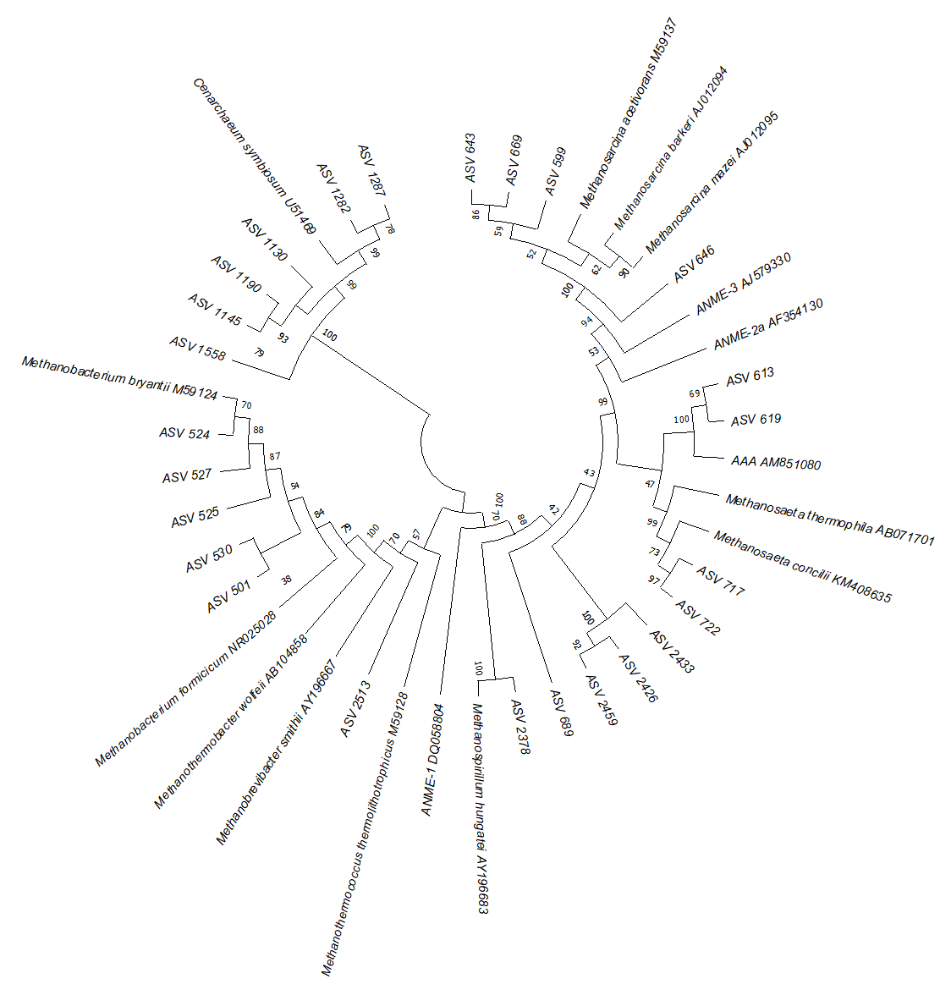


**Fig. S5.** Phylogenetic tree of ASVs co-occurrence with *Bathyarchaeia*. The Phylogenetic tree was built using Maximum Likelihood in Mega 11.
